# Mortality Associated with Neurofibromatosis 1: A Cohort Study of 1895 Patients in 1980-2006 in France

**DOI:** 10.1186/1750-1172-6-18

**Published:** 2011-05-04

**Authors:** Tu Anh Duong, Emilie Sbidian, Laurence Valeyrie-Allanore, Cédric Vialette, Salah Ferkal, Smaïl Hadj-Rabia, Christophe Glorion, Stanislas Lyonnet, Michel Zerah, Isabelle Kemlin, Diana Rodriguez, Sylvie Bastuji-Garin, Pierre Wolkenstein

**Affiliations:** 1 AP-HP, Hôpital Henri-Mondor, Service de Dermatologie, F-94010 Créteil, France; 2 Université Paris Est, F-94010 Créteil, France; 3 AP-HP, Hôpital Henri-Mondor, Pôle Recherche Clinique-Santé Publique, F-94010 Créteil, France; 4 AP-HP, Hôpital Henri-Mondor, Centre de référence des Neurofibromatoses, F-94010 Créteil, F-94010, France; 5 Université Paris Est, LIC EA4393 (Laboratoire d'Investigation Clinique), F-94010 Créteil, France; 6 Unité Recherche Clinique, AP-HP, Hôpital Henri-Mondor, F-94010 Créteil, France; 7 Université Paris Est, INSERM, Centre d'Investigation Clinique 006, F-94010 Créteil, France; 8 AP-HP, Hôpital Necker-Enfants Malades, Service de Dermatologie, Centre de référence des Maladies Génétiques à Expression CutanéeF-75743 Paris, Université Paris-Descartes, France; 9 AP-HP, Hôpital Necker-Enfants Malades, Service d'Orthopédie, F-75743 Paris, France; 10 AP-HP, Hôpital Necker-Enfants Malades, Service de Génétique, F-75743 Paris, France; 11 AP-HP, Hôpital Necker-Enfants Malades, Service de Neurochirurgie, Paris F-75743, France; 12 AP-HP, Hôpital Trousseau, Service de Neuropédiatrie, Paris F-75571, France; 13 Université Pierre et Marie Curie, Paris, France

## Abstract

**Background:**

Neurofibromatosis 1 (NF1), a common autosomal dominant disorder, was shown in one study to be associated with a 15-year decrease in life expectancy. However, data on mortality in NF1 are limited. Our aim was to evaluate mortality in a large retrospective cohort of NF1 patients seen in France between 1980 and 2006.

**Methods:**

Consecutive NF1 patients referred to the National French Referral Center for Neurofibromatoses were included. The standardized mortality ratio (SMR) with its 95% confidence interval (CI) was calculated as the ratio of observed over expected numbers of deaths. We studied factors associated with death and causes of death.

**Results:**

Between 1980 and 2006, 1895 NF1 patients were seen. Median follow-up was 6.8 years (range, 0.4-20.6). Vital status was available for 1226 (65%) patients, of whom 1159 (94.5%) survived and 67 (5.5%) died. Overall mortality was significantly increased in the NF1 cohort (SMR, 2.02; CI, 1.6-2.6; *P *< 10^-4^). The excess mortality occurred among patients aged 10 to 20 years (SMR, 5.2; CI, 2.6-9.3; *P *< 10^-4^) and 20 to 40 years (SMR, 4.1; 2.8-5.8; *P *< 10^-4^). Significant excess mortality was found in both males and females. In the 10-20 year age group, females had a significant increase in mortality compared to males (SMR, 12.6; CI, 5.7-23.9; and SMR, 1.8; CI, 0.2-6.4; respectively). The cause of death was available for 58 (86.6%) patients; malignant nerve sheath tumor was the main cause of death (60%).

**Conclusions:**

We found significantly increased SMRs indicating excess mortality in NF1 patients compared to the general population. The definitive diagnosis of NF1 in all patients is a strength of our study, and the high rate of death related to malignant transformation is consistent with previous work. The retrospective design and hospital-based recruitment are limitations of our study. Mortality was significantly increased in NF1 patients aged 10 to 40 years and tended to be higher in females than in males.

## Background

Neurofibromatosis 1 (NF1; MIM#162200) is an inherited autosomal dominant disorder with an incidence of 1 in 2500-3000 births [1]. NF1 is fully penetrant by 8 years of age. According to the National Institutes of Health (NIH), the diagnosis of NF1 requires at least two of the following seven criteria: six or more café-au-lait spots measuring at least 5 mm in prepupertal patients and 15 mm in postpubertal patients, multiple axillary freckles, two or more neurofibromas (NFs) of any type or one plexiform neurofibroma, Lisch hamartomas, optic pathway glioma, bone dysplasia, and at least one affected first-degree relative [2]. The phenotype of NF1 varies substantially across patients.

The *NF1 *gene on chromosome 17q11.2 encodes the tumor suppressor protein neurofibromin. Loss of this protein is associated with an increased risk of developing tumors [3]. In a 12-year follow-up study of 70 adult NF1 patients in Sweden, life expectancy was decreased by 15 years compared to the general population, and malignancy was the main cause of death [4]. A 1986 study in a Danish cohort of 212 patients diagnosed 42 years earlier showed excess mortality with an increase in malignancies in the males but not in the females, compared to the general population [5]. Several clinical features such as internal or subcutaneous NFs have been shown to predict mortality in NF1 patients [6,7]. In a cohort of 448 NF1 patients in the UK, the overall risk of cancer was increased 2.7-fold compared to the general population, and malignant peripheral nerve sheath tumor (MPNST) was the leading cause of death [8]. A proportional mortality study based on death certificate data in the US from 1983 to 1997 demonstrated that NF1 patients were 34 times more likely to have a malignant connective or soft tissue neoplasm listed on their death certificate than individuals without NF1 persons (proportional mortality rate, 34.3; 95% confidence interval [95%CI], 30.8-38) [9]. This study also showed an about 15-year decrease in life expectancy in NF1 patients [9].

Nevertheless, data on mortality in NF1 are limited. Our aim was to evaluate mortality in a large cohort of patients with NF1 seen in France between 1980 and 2006. We computed standardized mortality ratios (SMRs). We evaluated risk factors for death and causes of death.

## Patients and Methods

### Definitions and study cohort

The study cohort was composed of consecutive patients meeting NIH criteria for NF1 [2] who were referred to the hospital departments of the Paris conurbation that constitute the National French Referral Center for Neurofibromatoses (dermatology departments at the Henri Mondor and Necker-Enfants Malades hospitals and neuropediatric department at the Trousseau hospital). We identified NF1 patients referred to these departments between January 1, 1980, and December 31, 2006, in the NF1 Network Database maintained by the National French Hospital Database (PMSI). Data from all sources were linked and compared to eliminate duplications and resolve discrepancies.

The study was approved by the Ile-de-France IX ethics committee, Paris, France. The study complied with Helsinki guidelines.

### Data collection

For each patient, age at the beginning of the study period, sex, and clinical features were abstracted from the medical charts and/or database. Detailed information was available on the cardinal dermatological features of NF1 (café-au-lait spots, freckles, Lisch nodules, and cutaneous and plexiform neurofibromas). We also recorded the following features as present or absent: orthopedic complications (scoliosis and pseudoathrosis), neurological abnormalities (hydrocephalus and high T2 signal intensity lesions on magnetic resonance imaging of the brain), and renal artery stenosis. We recorded all available data on tumors known to occur frequently in NF1 patients (central nervous systems tumors, MPNSTs, and pheochromocytomas).

### Mortality assessment

**Figure 1:**
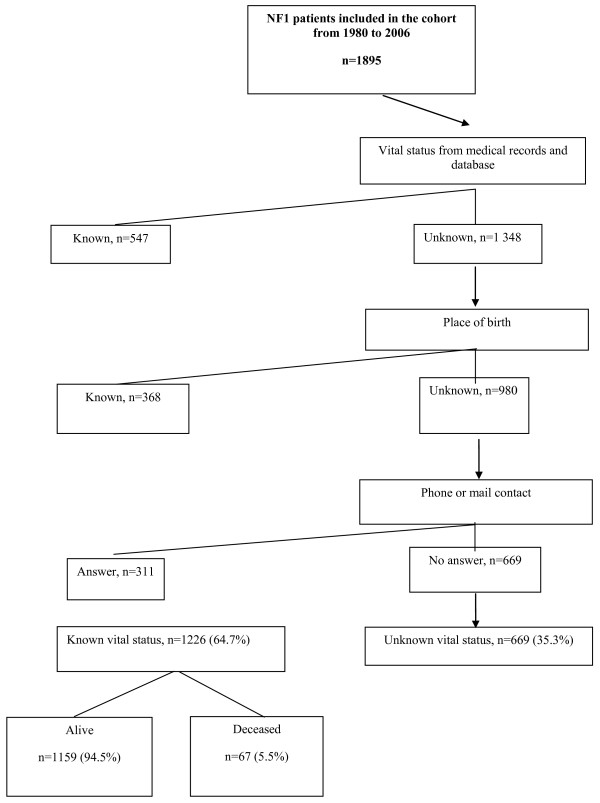
**Flow chart of the vital status of patients with NF1 cohort seen between 1980 and 2005**.

Our goal was to determine vital status as of December 31, 2006. We first consulted the medical records and network database. Then, we searched the National French Mortality Database (INSERM CépiDC) for information on patients whose place of birth was known; we checked this information by calling the town hall of the place of birth. Finally, we sought to contact patients by phone and mail. Patients whose vital status was still unknown after these three steps were classified as having an unknown vital status (unknownVS group) and other patients as having a known vital status (knownVS group) (Figure 1).

### Statistical analysis

Data were double entered and analyzed using STATA software version 11 (Stata Corporation, College Station, TX). Quantitative variables are reported as median (range) and qualitative variables as number (%).

We compared the unknownVS and knownVS groups using univariate analysis (chi-square test, or Fisher exact test where appropriate, and nonparametric Mann-Whitney test). Because age influences the prevalence and number of NF1 features as café-au-lait spots and neurofibromas, we computed age-adjusted odds ratios (aORs) with their 95% confidence intervals (95%CIs) separately for each variable. In the knownVS group, we computed the standardized mortality ratio (SMR) as the ratio of observed over expected deaths. SMR values greater than 1 with a CI that does not include 1 indicate excess mortality. The expected number of deaths was obtained by applying the mortality rates in France from 1980 to 2006 (by 5-year age groups and 5-year calendar periods, separately in females and males) to the appropriate person-years in the cohort. We considered the following age groups: younger than 10 years, 10-20 years, 20-40 years, 40-60 years, and 60 years or older. The 95%CI of the SMR was computed assuming a Poisson distribution [10]. The chi-square test of dispersion of the Poisson distribution was calculated. Sensitivity analyses were performed. First, to avoid overestimation of mortality, we computed the SMR for the overall cohort, assuming that patients in the unknownVS group were alive on December 31, 2006. Second, to minimize survivorship bias (bias related to underestimation of mortality in childhood due to patient attrition), we computed the SMR for patients who were born after January 1, 1980, the beginning of our study period.

## Results

### Study cohort

Between January 1, 1980, and December 31, 2006, 1895 NF1 patients were seen at the National French Referral Center for Neurofibromatoses. Their median follow-up was 6.8 years [0.4-20.6]. At inclusion, median age was 17.7 [0-76] years, and 549 (44.8%) patients were younger than 18 years (Table 1). At the end of the study period, median age was 25.9 years [2-82].

**Table 1:** Characteristics of the NF1 patients according to whether their vital status was known or unknown

Clinical features*	KnownVS group n = 1226 No (%)†	UnknownVS group No (%)†	OR (95%CI) ‡	*P *value
Age, years (n = 1893)	25.9 [2-82]□	25.4 [0.2-68]□	-	0.19
Male gender (n = 1 894)	579 (47)	304 (46)	0.9 (0.8-1.1)	0.57
Familial case (n = 1382)	515 (57)	278 (57)	1.0 (0.8-1.3)	0.95
At least 6 café-au-lait spots (n = 1889)	1 046 (86)	584 (87)	0.9 (0.7-1.2)	0.56
Freckles (n = 1 871)	1 043 (86)	509 (78)	1.7 (1.3-2.2)	<10^-4^
Neurofibromas (n = 1879)	923 (76)	495 (75)	1.0 (0.8-1.3)	0.87
Plexiform neurofibromas (n = 1865)	361 (30)	175 (27)	1.2 (0.9-1.4)	0.20
Lisch nodules (n = 1463)	382 (40)	204 (40)	1.1 (0.8-1.2)	0.87
Renal artery stenosis (n = 1361)	5 (1)	3 (1)	0.7 (0.2-3.1)	0.70
Scoliosis (n = 1373)	231 (25)	104 (24)	1.1 (0.8-1.4)	0.72
Nonunion (n = 1835)	45 (4)	20 (3)	1.2 (0.7-2.0)	0.57
Hydrocephalus (n = 1827)	32 (3)	23 (4)	0.7 (0.4-1.2)	0.22
OBNI (n = 801)	210 (40)	105 (38)	1.0 (0.7-1.4)	0.96
Optic pathway glioma (n = 1812)	161 (14)	69 (11)	1.3 (0.9-1.7)	0.11
MPNSTs (n = 1842)	80 (7)	16 (3)	2.7 (1.6-4.7)	<10^-4^
Pheochromocytoma (n = 1836)	13 (1)	7 (1)	1.3 (0.5-3.7)	0.61

Abbreviations: VS, vital status; OR, odds ratio; 95%CI, 95% confidence interval; OBNI, high T2-signal intensity lesions on magnetic resonance imaging of the brain; MPNST, malignant peripheral nerve sheath tumor

* Values in parentheses are the numbers of patients for whom data were available.

□Age at the end of the study period in patients whose vital status was known and age at last follow-up in those whose vital status was unknown status

† Percentages may not total 100, because of missing data.

‡The ORs were adjusted for age.

Data are numbers (%) or median [range].

Vital status was known for 1226 (65%) of the 1895 patients (Figure 1). At last follow-up, patients in the unknownVS group had a median age of 25.4 years [0.2-68] (Table 1). Compared to the unknownVS group, the knownVS group had significantly higher prevalence of freckles (aOR,1.7; 95%CI, 1.3-2.2) and MPNSTs (aOR, 2.7; 95%CI, 1.6-4.7) (Table 1). The prevalence of optic pathway gliomas was nonsignificantly higher in the knownVS group (OR, 1.3; 95% CI, 0.9-1.7; *P *= 0.11).

Of the 1226 patients in the knownVS group, 1 159 (94.5%) survived and 67 (5.5%) died.

### Causes of death

The median age at death was 31.7 years [0-79.9], and 46.3% of patients who died were males. We did not reported any death from the same family. Compared to survivors, nonsurvivors had non significantly fewer café-au-lait spots (aOR, 0.6; 95%CI, 0.3-1.1]) and significantly more neurofibromas (aOR, 3.1; 95%CI, 1.2-8.4), plexiform neurofibromas (aOR, 1.8; 95%CI, 1.1-3.0), MPNSTs (aOR, 31.2; 95%CI, 17.4-55.9,) and pheochromocytomas (aOR, 4.3; 95%CI, 1.1-16.3; Table 2).

**Table 2:** Characteristics of the survivors and nonsurvivors among the 1226 NF1 patients whose vital status was known

Clinical features*	Survivors n = 1159 **No (%)**†	Nonsurvivors n = 67 **No (%)**†	OR **(95%CI**)‡	*P *value*
Age, years, median [range]	25.5 [2-74]	31.7 [0-80]	-	0.008
Male gender	548 (47)	31 (46)	1.0 (0.6-1.7)	0.98
Familial case (n = 897)	498 (58)	17 (53)	0.7 (0.3-1.4)	0.62
At least 6 café-au-lait spots (1220)	998 (86)	48 (74)	0.6 (0.3-1.1)	0.07
Freckles (n = 1217)	985 (86)	58 (88)	1.1 (0.5-2.4)	0.74
Neurofibromas (n = 1215)	863 (75)	60 (90)	3.1 (1.2-8.4	0.02
Plexiform neurofibromas (n = 1209)	333 (29)	28 (42)	1.8 (1.1-3.0)	0.02
Lisch nodules (n = 954)	366 (40)	16 (40)	0.9 (0.5-1.9)	0.89
Renal artery stenosis (n = 601)	5 (1)	0 (0)	-	0.60
Scoliosis (n = 934)	222 (25)	9 (18)	0.6 (0.3-1.3)	0.23
Nonunion (n = 1 194)	43 (4)	2 (3)	0.7 (0.2-3.2)	0.70
Hydrocephalus (n = 1 190)	31 (3)	1 (2)	0.6 (0.1-4.4)	0.60
OBNI (n = 526)	202 (40)	8 (30)	0.6 (0.3-1.5)	0.30
Optic pathway glioma (n = 1 172)	151 (13)	11 (17)	1.2 (0.6-2.6)	0.58
MPNST (n = 1198)	44 (4)	36 (55)	31.2 (17.4-55.9)	<10^-4^
Pheochromocytoma (n = 1193)	10 (1)	3 (5)	4.3 (1.1-16.3)	0.03

Abbreviations: OR, odds ratio; 95%CI, 95% confidence interval; OBNI, high T2-signal intensity lesions on magnetic resonance imaging of the brain; MPNST, malignant peripheral nerve sheath tumor

*Values in parentheses are the numbers of patients for whom data were available.

†Percentages may not total 100, because of missing data.

‡The ORS were adjusted for age.

Data are numbers (%) or median [range].

The cause of death was known for 58 (86.6%) patients and was related to NF1 in 56 (96.6%) of them. These fatal complications of NF1 included MPNSTs (n = 33, 60%), central nervous system tumors (n = 8, 14%), spinal cord compression by neurofibromas (n = 2, 3%), organ compression by neurofibromas (n = 5, 9%), and pheochromocytomas (n = 2, 3%).

### Standardized mortality ratios

Compared to the general population, overall mortality was significantly increased in the NF1 cohort (SMR, 2.02; 95%CI, 1.6-2.6; *P *< 10^-4^). The excess mortality was found in two age groups, 10-20 years (SMR, 5.2; 95%CI, 2.6-9.3; *P *< 10^-4^) and 20-40 years (SMR, 4.1; 95%CI, 2.8-5.8; *P *< 10^-4 ^; Table 3). Significant excess mortality was observed in both males (SMR, 1.8; 95% CI, 1.2-2.5; *P *= 0.01) and females (SMR, 2.9; 95%CI: 2.0-4.0; *P *< 10^-4 ^; Table 4). In the 10-20 year age group, mortality is higher in females (SMR, 12.6; 95%CI, 5.7-23.9) than in males (SMR, 1.8; 95%CI: 0.2-6.4).

**Table 3:** Number of deaths (n) and SMRs among the NF1 patients by age group

Age, years	Person- Years	Observed Deaths	Expected Deaths	SMR	95%CI
<10	11 905.5	4	4.2	0.95	0.3-2.4
10 to <20	8875	11	2.1	5.2	2.6-9.3
20 to <40	10 395	31	7.6	4.1	2.8-5.8
40 to <60	3657.5	11	11.6	0.9	0.5-1.7
≥60	480	10	7.7	1.3	0.6-2.4

SMR, standardized mortality ratio (observed deaths/expected deaths); 95%CI, 95% confidence interval

**Table 4:** Number of deaths (n) and SMRs among the NF1 patients, by age categories (years), by sex

	Male					Female				
**Age, years**	**Person- Years**	**Observed Deaths**	**Expected Deaths**	**SMR**	**95%CI**	**Person- Years**	**Observed Deaths**	**Expected Deaths**	**SMR**	**95%CI**

<10	5569.5	1	1.8	0.6	0.01-3.1	6336	3	1.8	1.7	0.3-4.8
10 to <20	4000	2	1.1	1.8	0.2-6.4	4875	9	0.7	12.6	5.7-23.9
20 to <40	4262.5	19	4.4	4.3	2.6-6.7	6132.5	12	2.6	4.7	2.4-8.2
40 to <60	1272.5	5	5.5	0.9	0.3-2.1	2385	6	4.8	1.3	0.5-2.7
≥60	162.5	4	4.8	0.8	0.2-2.1	317.5	6	2.7	2.3	0.8-4.9

Assuming that patients in the unknownVS group were alive at last follow-up did not substantially change the excess mortality in patients aged 10 to 20 years (SMR, 3.3; 95%CI, 1.6-5.8; *P *= 0.01) or 20 to 40 years (SMR, 2.4; 95%CI, 1.6-3.4; *P *= 0.001). When we confined the analysis to the 633 patients born after January 1, 1980, we found excess mortality in patients aged 10 to 30 years (SMR, 3.9; 95%CI, 2.3-6.3; *P *< 10^-4^). The excess mortality in this subgroup seemed higher than in the overall cohort.

## Discussion

Significant excess mortality was found in our overall cohort and in the subgroups aged 10 to 20 and 20 to 40 years. We did not find excess mortality in the subgroups aged more than 40. It could be related to lack of power due to a very low number of persons over 60. Excess mortality was significant in both females and males; in the 10-20 year age group, excess mortality was significantly higher in the females than in the males. The vast majority of deaths were due to malignancies and other complications of NF1.

We retrospectively identified consecutive NF1 patients referred to the French National Referral Center for Neurofibromatoses. An important strength of our study is that all patients had a definitive diagnosis of NF1. Furthermore, the overall prevalence of NF1 manifestations in our cohort was consistent with previous reports, [11] indicating that our cohort was representative of the NF1 population. We used multiple sources of information to determine vital status. Nevertheless, vital status was known for only about two-thirds of the patients, which may have resulted in information bias. However, to avoid overestimating mortality, we conducted an analysis assuming that all patients whose vital status was unknown were alive at last follow-up. We again found significant excess mortality among NF1 patients aged 10 to 40 years compared to the general population. Another limitation of our study is the retrospective design, which may have resulted in survivorship bias with underestimation of mortality due to higher values for both patient attrition and mortality in the older patients. However, when we conducted an analysis confined to patients born after the beginning of our study period, we still found significant excess mortality compared to the general population. Finally, our hospital-based recruitment may have led to a bias toward patients with more severe forms of NF1 and, therefore, to overestimation of the SMR. However, our findings are consistent with previous reports [4,5,9].

Among the 58 patients who died and in whom the cause of death was known, 56 died from NF1 complications, chiefly malignancies (MPNSTs in 60% and cerebral tumors in 14%). Our results are consistent with those of a prospective study of the incidence of cancer in NF1 patients in the UK [8]. In this study, the overall risk of cancer was 2.7 times higher in NF1 patients than in the general population, and increases were found in central nervous system tumors and connective tissue tumors, as well as in breast cancer in women younger than 50 years (standardized incidence ratio, 4; 95%CI, 1.1-10.3). The risk of mortality related to breast cancer was not reported. In our study, we did not assess the prevalence of breast cancer, which was not the reported cause of death in any of the patients. Similarly, in a death certificate study done in the US, NF1 patients were 34 times more likely to have a malignant connective or other soft tissue neoplasm listed on their death certificate than patients without NF1 [9]. The proportionate mortality ratios for malignancies were 6.07 for patients who died between 10 and 19 years of age and 4.93 for those who died between 20 and 29 years of age. Proportionate mortality ratios were also increased for central nervous system tumors and vascular disease. The authors concluded that the impact of NF1 on mortality from malignancies and vascular disease was focused on patients younger than 40 years of age [9]. In our cohort, cardiovascular disease was not reported as a cause of death. Our chart review did not assess cardiovascular morbidity and we also did not estimate the cardiovascular risk in NF1 patients.

In our study, MPNSTs were the leading cause of death (n = 33, 60%). NF1 is widely recognized as a risk factor for MPNSTs, and the lifetime risk of MPNSTs in NF1 patients has been estimated at 8%-13% [1,12]. In an earlier study, we also found that MPNST was the main cause of death and that risk factors for MPNST were the absence of cutaneous neurofibromas, presence of at least two subcutaneous neurofibromas, and facial asymmetry [6]. Further studies confirmed that subcutaneous neurofibromas predicted mortality (OR, 3.6; 95%CI, 1.2-11.3) [7] and that internal plexiform neurofibromas were strongly associated with MPNST (OR, 18.06; 95%CI, 4.55-73.4) [13]. We did not have detailed information on these features in our patients and were therefore unable to assess their potential relationship to mortality. Nonsurvivors had fewer café-au-lait spots than did survivors. Furthermore, excess mortality was confined to patients aged 10 to 40 years. These two variables are among the four used in the NF1 score for predicting internal neurofibromas (age ≤30 years, fewer than six café-au-lait spots, no cutaneous neurofibromas, and two or more subcutaneous neurofibromas) [14]. Internal neurofibromas are strongly associated with MPNSTs[14].

In our study, median age at death was 31.7 years [0-77.9], that is, considerably younger than in previous studies. However, our patients were young, with a median age of 25.9 years at the time of the analysis. In a Japanese death certificate study, mean age at death from neurofibromatosis was 43 years, but NF1 and NF-2 were not clearly separated [15]. In a death certificate study done in the US, mean age at death in NF1 patients was 54.4 years, that is, 15.7 years younger than in the general population [9]. In this study, the authors used multiple-cause mortality files compiled from US death certificates and identified 3770 cases of presumed NF1 among the 32 722 122 deaths recorded from 1983 to 1997 [9].

Mean age at death in our study was lower in females than males, in keeping with an earlier study [5]. We also found that excess mortality was significantly greater in females than in males between 10 and 20 years of age. Sex hormones may affect the NF1 phenotype. Immunostaining studies identified the progesterone receptor in neurofibromas [16]. Furthermore, orthotopic xenografts of an immortal human NF1-derived Schwann cell line into the sciatic nerves of female mice with severe combined immunodeficiency produced MPNSTs, and tumor cell proliferation was decreased in ovariectomized mice and restored by estrogen or progesterone replacement therapy [17]. Additional experiments by the same group supported a role for estrogen and progesterone on the growth of MPNST obtained by xenografting [18]. Finally, in vitro and in vivo studies on MPNST cell lines or MPNST xenografts showed that tamoxifen inhibited malignant cell growth in an estrogen-receptor-independent manner [1,19].

In conclusion, our evaluation of SMRs indicates significant excess mortality in NF1 patients compared to the general population. Although the retrospective design and hospital-based recruitment are limitations of our study, the predominance of cancer among causes of death is consistent with earlier work. We found that excess mortality was confined to the 10-40 year age group and that females in the 10-20 year age group had significantly greater excess mortality than males. Additional studies following our cohort ageing might accurately assess mortality above 40 years in NF1 patients.

## Abbreviations

NF1: Neurofibromatosis 1; NF-2: Neurofibromatosis 2; NFs: Neurofibromas; MPNST: Malignant peripheral Nerve sheath tumor; CI: Confident Interval; aOR: adjusted Odds Ratio; SMR: Standardized mortality ratios

## Competing interests

The authors declare that they have no competing interests.

## Authors' contributions

SBG, PW and LA have made substantial contributions to conception and design. SBG, PW, TAD, SF, CV and ES have made substantial contributions to acquisition of data, or analysis and interpretation of data. SBG, PW, TAD and ES have been involved in drafting the manuscript or revising it critically for important intellectual content. All the authors have given final approval of the version submitted for publication.

## References

[B1] Huson SM Compston DA Clark P Harper PSI A genetic study of von Recklinghausen neurofibromatosis in south east Wales Prevalence, fitness, mutation rate, and effect of parental transmission on severity J Med Genet 1989 26 704 11 10.1136/jmg.26.11.704 2511318 PMC1015740

[B2] National Institutes of Health Consensus Development Conference Statement: neurofibromatosis. Bethesda, Md., USA, July 13-15, 1987 Neurofibromatosis 1988 1 172 8 3152465

[B3] Shen MH Harper PS Upadhyaya M Molecular genetics of neurofibromatosis type 1 (NF1) J Med Genet 1996 33 2 17 10.1136/jmg.33.1.2 8825042 PMC1051805

[B4] Zoller M Rembeck B Akesson HO Angervall L Life expectancy, mortality and prognostic factors in neurofibromatosis type 1. A twelve-year follow-up of an epidemiological study in Goteborg, Sweden Acta Derm Venereol 1995 75 136 40 7604643 10.2340/0001555575136140

[B5] Sorensen SAMJ Nielsen A Long-term follow-up of von Reckilinghausen neurofibromatosis. Survival and malignant neoplams N England J Med 1986 17 1010 5 10.1056/NEJM198604173141603 3083258

[B6] Khosrotehrani K Bastuji-Garin S Zeller J Revuz J Wolkenstein P Clinical risk factors for mortality in patients with neurofibromatosis 1: a cohort study of 378 patients Arch Dermatol 2003 139 187 91 10.1001/archderm.139.2.187 12588224

[B7] Khosrotehrani K Bastuji-Garin S Riccardi VM Birch P Friedman JM Wolkenstein P Subcutaneous neurofibromas are associated with mortality in neurofibromatosis 1: a cohort study of 703 patients Am J Med Genet A 2005 132A 49 53 10.1002/ajmg.a.30394 15523617

[B8] Walker LTD Easton D Ponder B Ponder M Frayling I Baralle D A prospective study of neurofibromatosis type 1 cancer incidence in the UK Br J Cancer 2006 17 233 8 10.1038/sj.bjc.6603227 PMC2360616 16786042

[B9] Rasmussen SA Yang Q Friedman JM Mortality in neurofibromatosis 1: an analysis using U.S. death certificates Am J Hum Genet 2001 68 1110 8 10.1086/320121 11283797 PMC1226092

[B10] Breslow NE Day NE Statistical methods in cancer research Volume II--The design and analysis of cohort studies IARC Sci Publ 1987 82 1 406 3329634

[B11] Friedman JM Birch PH Type 1 neurofibromatosis: a descriptive analysis of the disorder in 1,728 patients Am J Med Genet 1997 70 138 43 10.1002/(SICI)1096-8628(19970516)70:2<138::AID-AJMG7>3.0.CO;2-U 9128932

[B12] Evans DG Baser ME McGaughran J Sharif S Howard E Moran A Malignant peripheral nerve sheath tumours in neurofibromatosis 1 J Med Genet 2002 39 311 4 10.1136/jmg.39.5.311 12011145 PMC1735122

[B13] Tucker T Wolkenstein P Revuz J Zeller J Friedman JM Association between benign and malignant peripheral nerve sheath tumors in NF1 Neurology 2005 65 205 11 10.1212/01.wnl.0000168830.79997.13 16043787

[B14] Sbidian EWP Valeyrie-Allanore L Rodriguez D Hadj-Rabia S Ferkal S Lacour JP Leanoard JC Taillandier L Sportich S Berbis P Bastuji-Garin S NF-1Score: a prediction score for internal neurofibromas in neurofibromatosis-1 J Invest Dermatol 2010 130 2173 8 10.1038/jid.2010.100 20428190

[B15] Imaizumi Y Mortality of neurofibromatosis in Japan, 1968-1992 J Dermatol 1995 22 191 5 7738275 10.1111/j.1346-8138.1995.tb03369.x

[B16] Overdiek AWU Mayatepek E Rosenbaum T Schwann cells from human neurofibromas show increased proliferation rates under the influence of progesterone Pediatr Res 2008 64:40 3 10.1203/PDR.0b013e31817445b8 18360307

[B17] Perrin GQLH Fishbein L Thomson SA Hwang MS Scarborough MT Yachnis AT Wallace MR Mareci TH Muir D An orthotopic xenograft model of intraneural NF1 MPNST suggests a potential association between steroid hormones and tumor cell proliferation Lab Invest 2007 87 1092 102 10.1038/labinvest.3700675 17876295

[B18] Li HZX Fishbein L Kweh F Campbell-Thompson M Perrin GQ Muir D Wallace M Analysis of steroid hormone effects on xenografted human NF1 tumor schwann cells Cancer Biol Ther 2010 15 758 64 10.4161/cbt.10.8.12878 PMC3093914 20699653

[B19] Byer SJEJ Brossier NM Clodfelder-Miller BJ Turk AN Carroll AJ Kappes JC Zinn KR Prasain JK Carroll SL Tamoxifen inhibits malignant peripheral nerve sheath tumor growth in an estrogen receptor-independant manner Neuro Oncol 2011 13 28 41 10.1093/neuonc/noq146 21075781 PMC3018903

